# Partial anomalous pulmonary venous connection to superior vena cava that overrides across the intact atrial septum and has bi-atrial connection in a 75-year-old female presenting with pulmonary hypertension

**DOI:** 10.1186/1471-2261-14-149

**Published:** 2014-10-25

**Authors:** Hong Wang, Hanxiong Guan, Dao Wen Wang

**Affiliations:** Division of Cardiology and Department of Internal Medicine, Tongji Hospital, Tongji Medical College of Huazhong University of Science and Technology, 1905 Jiefang Dadao, Wuhan, 430030 PR China; Department of Radiology, Tongji Hospital, Tongji Medical College of Huazhong University of Science and Technology, 1905 Jiefang Dadao, Wuhan, 430030 PR China

**Keywords:** Sinus venosus atrial defect (SVD), Partial anomalous pulmonary venous connection (PAPVC), Pulmonary hypertension

## Abstract

**Background:**

Partial anomalous venous connection (PAPVC) is a rare congenital heart disease where the blood flow from one or more pulmonary veins (but not all) returns to the right atrium or systemic venous circulation and is often associated with a sinus venosus atrial defect (SVD). Transthoracic echocardiography (TTE) can provide limited information for this anomaly and the diagnosis of this congenital defect has been a clinical challenge.

**Case presentation:**

We report here a case of a 75-year-old female with adult-onset pulmonary arterial hypertension (PAH), hypoxemia and right-sided chamber dilatation. The diagnosis of PAPVC was made incidentally by multidetector computed tomographic angiography (MCTA) that was performed to exclude pulmonary embolism. In this type of PAPVC, the atrial septum is intact, the right upper pulmonary vein (RUPV) connects to the superior vena cava (SVC), and the SVC overrides across the atrial septum and has bi-atrial connection, all of which are clearly manifested by MCTA.

**Conclusions:**

This case indicates the need to exclude a PAPVC and SVD in unexplained pulmonary hypertension, and MCTA is a reliable non-invasive imaging technique with high resolution and wide anatomic coverage. The case also demonstrates that the coexisting SVD with PAPVC is an anomalous venous connection instead of atrial septal defect (ASD) and its key feature is the overriding of SVC or IVC across the intact atrial septum.

## Background

Partial anomalous venous connection (PAPVC) is defined as one or more, but not all, pulmonary veins drain into the right atrium or a systemic vein instead of the left atrium as in the normal heart. One of the most common types of PAPVC is the one in which the right upper pulmonary vein (RUPV) connects to the right atrium or the superior vena cava (SVC) and that often coexists with a sinus venosus atrial defect (SVD) [1, 2]. However, the anatomical features of SVD, whether it is an atrial septal defect (ASD) or an anomalous venous connection, have been a matter of debate [3, 4].

Herein we report a 75-year-old woman with unexplained pulmonary arterial hypertension (PAH) and right-sided chamber dilatation. The presence of PAPVC and SVD was confirmed by multidetector computed tomographic angiography (MCTA) that showed the anatomical features of this congenital defect, including the RUPV connecting to the SVC, atrial septum being intact, and the SVC overriding the intact atrial septum with bi-atrial connection. These features demonstrated the coexisting SVD with PAPVC was an anomalous venous connection instead of ASD.

## Case presentation

A 75-year-old woman was presented to our hospital with 10 years of mild exertional dyspnea and palpitation. She didn’t see any doctors until 1 month before presentation, when the symptoms of dyspnea and palpitation aggravated. She was found to have cardiomegaly and left lung infection by chest radiograph, and a presumptive diagnosis of dilated cardiomyopathy was made in a local hospital. Diuretics and inotropic agents were given but provided no relief of symptoms. She was then referred to our hospital for further evaluation.

**Figure 1:**
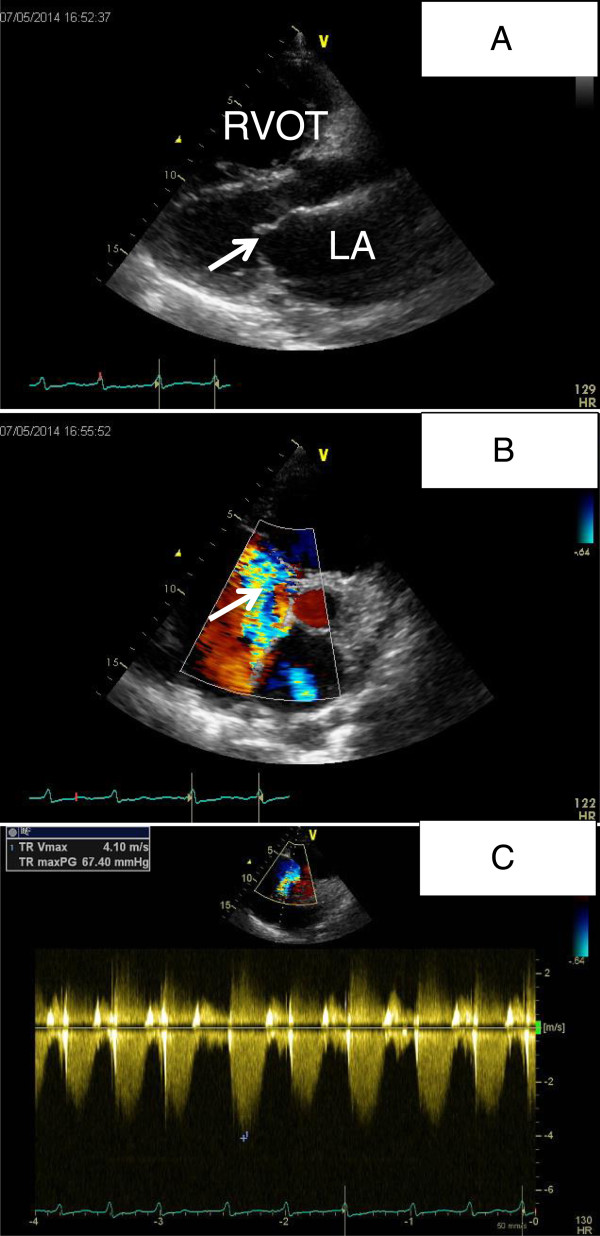
**TTE revealed PAH with right-sided chamber enlargement. A**: Dilatation of the RVOT(right ventricular outflow tract) was shown. **B**: Severe tricuspid regurgitation was shown in the parasternal short axis view (white arrow) with dilatation of RV and RA. **C**: The pressure gradient between the RV and RA was around 67 mmHg by measuring the tricuspid regurgitation Doppler jet.

On admission the patient’s blood pressure was 121/68 mmHg, heart rate was around 110 bpm and irregular. The jugular veins were distended. Cardiac examination revealed an irregular tachycardia and a systolic apical murmur of grade 2/6. Rales were heard over the left lung base. The liver edge was palpable below the costal margin with tender. Edema was present in the both lower extremities. Laboratory tests revealed unremarkable findings except for the reduced blood oxygen pressure of 57 mmHg. Electrocardiogram showed atrial fibrillation and evidence of right ventricular hypertrophy. Transthoracic echocardiography (TTE) showed PAH with the estimated pulmonary arterial systolic pressure of 77 mmHg and dilatation of the right ventricle (RV) and right atrium (RA) (Figure 1A, B, C). Transesophageal echocardiography (TEE) was unable to complete successfully.

**Figure 2:**
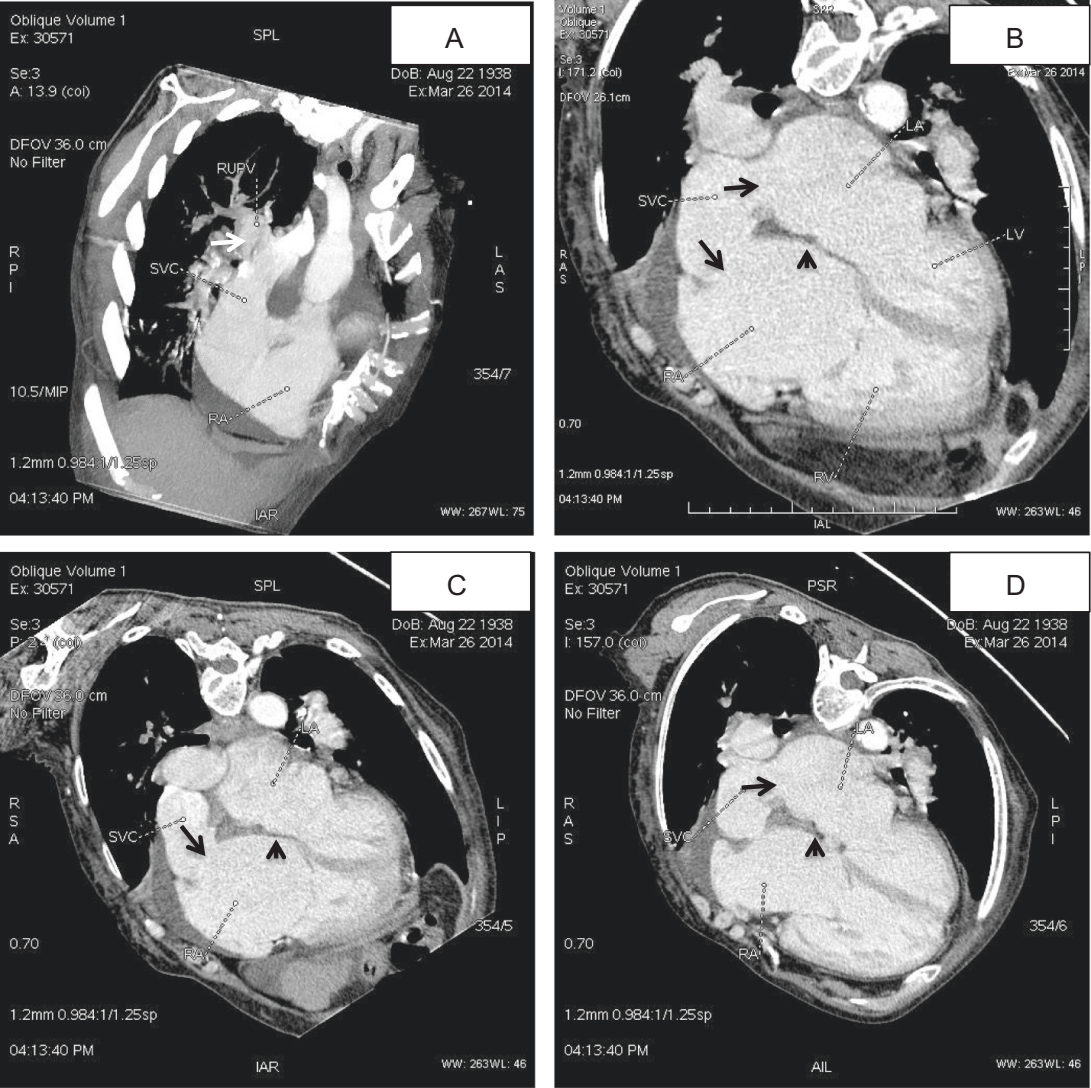
**MCTA demonstrated a PAPVC associated with a SVD. A**: The RUPV was unroofed and drained into the SVC at the level of the caval atrial junction (white arrow). **B**: The SVC had bi-atrial connection and overrode across the atrial septum (black arrow), and the atrial septum was actually intact (black triangle). **C**: The SVC had one opening connecting to the right atrium (black arrow); **D**: The SVC had another opening connecting to the left atrium (black arrow).

Pulmonary embolism was suspected and MCTA was performed then, which confirmed the diagnosis of PAPVC and SVD. The anatomical and morphological features of this congenital defect were clearly revealed by MCTA. The RUPV was unroofed and drained into the SVC at the level of the caval atrial junction (Figure 2A). The atrial septum was actually intact and no true atrial ASD was present (Figure 2B, C, D). The SVC overrode across the intact atrial septum and had bi-atrial connection (Figure 2B) with one opening connecting to the right atrium (Figure 2C) and another opening to the left atrium (Figure 2D).

Right heart catheterization and surgery correction of the anomaly was suggested but was declined by the patient. Intravenous infusion of Alprostadil was initiated with 20 μg twice daily and then Beraprost with 40 μg three times daily. Sildenafil was recommended but declined by the patient due to the high cost. The patient was discharged a week later with improved 6-Minute Walking Distance (300 meters at baseline and 320 meters at the day of discharge).

## Discussion

PAPVC is a rare congenital heart disease, with a prevalence of 0.1-0.2% in adult population reported by a recent study [1]. Anomalous right-sided pulmonary veins could return directly to the right atrium or a systemic vein such as SVC, inferior vena cava (IVC), azygos vein, hepatic vein or portal vein. The left-sided PAPVC might drain into the innominate vein, coronary sinus and hemiazygos vein [1, 5]. The RUPV connecting to the SVC is one of the most common forms of PAPVC and is often associated with a SVD [2, 5, 6].

The diagnosis of PAPVC has been a clinical challenge. The patient’s clinical manifestation and progression vary significantly depending on the amount of the intra-cardiac shunt. Symptoms or signs as dyspnea, atrial arrhythmias, right heart failure, and pulmonary hypertension may occur but are not specific to PAPVC. A great number of PAPVC cases were misdiagnosed as primary pulmonary hypertension [7], or were diagnosed incidentally [1, 8]. In our case, the patient developed the symptoms of PAH in her sixth to seventh decades. No intra-cardiac shunt was found by TTE. MCTA was then performed to exclude pulmonary embolism, which incidentally revealed the presence of PAPVC and the associated SVD.

Although TTE is less sensitive and may miss around 33% patients [9], it remains the first choice of non-invasive imaging modality to diagnose PAPVC. TTE could provide useful information other than direct evidence of intra-cardiac shunts. Any findings by TTE with unexplained PAH or right-sided chamber enlargement should warrant further studies as TEE or MCTA. TEE has better sensitivity and is highly diagnostic for PAPVC in experienced operators [5]. Other methods as MCTA and cardiac MRI have gained increasingly importance for non-invasive detection of vascular anomalies [1, 10]. In our opinion, contrast-enhanced MCTA has high spatial and temporal resolution and is less operator-dependent compared with TEE. As shown in our case, the anatomical features as the presence, number of anomalous veins and the associated defects can be reliably discovered by MCTA.

SVD is the most common congenital anomaly associated with PAPVC. A lot reports had simply indicated that this anomaly is a sinus venosus type of ASD but failed to study the accurate anatomical or morphological features of SVD [5, 10, 11]. Recently, a few reports argued the presence of ASD in SVD [3, 4]. Oliver et al. studied the anatomical features of SVD by comparing TEE data with surgical findings in 24 patients with a posterior inter-atrial communication closely related to the orifice of SVC or IVC. Their findings supported that SVD should be regarded as an anomalous venous connection with an inter-atrial communication outside the confines of the atrial septum [4]. The MCTA imaging results in our case are consistent with their findings. Therefore, the key anatomical feature of SVD is overriding of the mouth of the SVC, as shown in our case, or IVC across the intact muscular border of the oval fossa, instead of ASD.

At the beginning of development, the primitive lung drains via the splanchnic venous plexus into the systemic venous circulation (cardinal and umbilical veins). The primitive PV or so-called PV Anlage originates from midpharyngeal endothelial strand (MPES), which is a single non-lumenized structure at the time, and connects to the sinus venosus segment of the heart. Additionally, the dorsal mesenchymal protrusion (DMP), located at the entrance of PV Anlage into the sinus venosus (connecting to the common atrium), contributes to the atrial septum. Between 4th and 5th week of development, the primitive PV lumenizes and connects to the LA and the primitive connections of the lung to the systemic veins regress. If PVs do not lumenize or become atretic, pulmonary-systemic communications persists and TAPVC (total anomalous venous connection) or PAPVC develop. At the same stage, if DMP contribution to atrial septation is impaired, a so-called sinus venosus defect develops [12, 13]. As to the SVC development, the anterior cardinal veins bring the blood into the left and right common cardinal vein. They then empty via the right and left sinus horn into the sinus venosus and then into the atrium. At the time of atrial septation, due to obliteration of the veins on the left side of embryo, the right sinus horn and veins greatly enlarge and the sinoatrial opening shifts to the right and opens into the future right atrium. The right anterior cardinal and right common cardinal veins become the SVC. The left sinus horn atrophies and the left common cardial vein greatly reduced to form the coronary sinus [12, 13]. Any defects with this process may cause the SVC connecting to both the right and left atria.

Therefore, the possible embryological explanation to the anomaly reported here is that the RUPV does not lumenize when it should and the corresponding lung segment drain its blood to the systemic venous circulation by means of persistence of connection to the SVC. At the same time, the DMP contribution to atrial septation is non-defective and the atrial septum is intact. At last, the right sinus horn and right veins do not shift and the left sinus horn and left veins do not regress properly, which cause bi-atrial connection of SVC.

## Conclusions

This case highlights the need to exclude a PAPVC and SVD in patients with unexplained pulmonary hypertension and right-sided chamber enlargement. MCTA is a reliable non-invasive imaging modality with high resolution and wide anatomic coverage. The case also demonstrates that the common coexisting defect with PAPVC, SVD, is actually an anomalous venous connection instead of ASD and its key feature is the overriding of SVC or IVC across the intact atrial septum.

## Consent

Written informed consent was obtained from the patient for publication of this Case report and any accompanying images. A copy of the written consent is available for review by the Editor of this journal.

## Abbreviations

PAPVC: Partial anomalous venous connection
TAPVC: Total anomalous venous connection
RUPV: Right upper pulmonary vein
SVC: Superior vena cava
SVD: Sinus venosus atrial defect
ASD: Atrial septal defect
PAH: Pulmonary arterial hypertension
RV: Right ventricle
RA: Right atrium
MCTA: Multidetector computed tomographic angiography
TTE: Transthoracic echocardiography
TEE: Transesophageal echocardiography
IVC: Inferior vena cava
MPES: Midpharyngeal endothelial strand
DMP: The dorsal mesenchymal protrusion
PV: Pulmonary vein.
